# A real time metabolomic profiling approach to detecting fish fraud using rapid evaporative ionisation mass spectrometry

**DOI:** 10.1007/s11306-017-1291-y

**Published:** 2017-11-02

**Authors:** Connor Black, Olivier P. Chevallier, Simon A. Haughey, Julia Balog, Sara Stead, Steven D. Pringle, Maria V. Riina, Francesca Martucci, Pier L. Acutis, Mike Morris, Dimitrios S. Nikolopoulos, Zoltan Takats, Christopher T. Elliott

**Affiliations:** 1 Institute for Global Food Security, Advanced ASSET Centre, School of Biological Sciences, Queen’s University Belfast, 18-30 Malone Road, Belfast, BT9 5BN Northern Ireland, UK; 2 Waters Research Centre, 7 Zahony Street, Budapest, 1031 Hungary; 3 Imperial College London, South Kensington Campus, Sir Alexander Fleming Building, London, SW7 2AZ UK; 4 Waters Corporation, Altrincham Road, Wilmslow, SK9 4AX UK; 5 Istituto Zooprofilattico Sperimentale del Piemonte Liguria e Valle d’Aosta, Via Bologna 148, 10154 Turin, Italy; 6 School of Electronics, Electrical Engineering and Computer Science, Queen’s University Belfast, Belfast, UK

**Keywords:** REIMS, Real time, No sample preparation, Fish, Species identification, Catch method

## Abstract

**Introduction:**

Fish fraud detection is mainly carried out using a genomic profiling approach requiring long and complex sample preparations and assay running times. Rapid evaporative ionisation mass spectrometry (REIMS) can circumvent these issues without sacrificing a loss in the quality of results.

**Objectives:**

To demonstrate that REIMS can be used as a fast profiling technique capable of achieving accurate species identification without the need for any sample preparation. Additionally, we wanted to demonstrate that other aspects of fish fraud other than speciation are detectable using REIMS.

**Methods:**

478 samples of five different white fish species were subjected to REIMS analysis using an electrosurgical knife. Each sample was cut 8–12 times with each one lasting 3–5 s and chemometric models were generated based on the mass range m/z 600–950 of each sample.

**Results:**

The identification of 99 validation samples provided a 98.99% correct classification in which species identification was obtained near-instantaneously (≈ 2 s) unlike any other form of food fraud analysis. Significant time comparisons between REIMS and polymerase chain reaction (PCR) were observed when analysing 6 mislabelled samples demonstrating how REIMS can be used as a complimentary technique to detect fish fraud. Additionally, we have demonstrated that the catch method of fish products is capable of detection using REIMS, a concept never previously reported.

**Conclusions:**

REIMS has been proven to be an innovative technique to help aid the detection of fish fraud and has the potential to be utilised by fisheries to conduct their own quality control (QC) checks for fast accurate results.

**Electronic supplementary material:**

The online version of this article (10.1007/s11306-017-1291-y) contains supplementary material, which is available to authorized users.

## Introduction

Economically motivated adulteration (EMA) of seafood products is a global issue occurring at alarmingly high rates (Table 1) with it estimated that on average 30% of commercial fish products sold are either misrepresented or mislabelled (Pardo et al. 2016). This equates to fraud in almost $120 billion of the global seafood industry as the Food and Agriculture Organisation of the United Nations (FAO) estimate the global seafood industry to be worth $400 billion annually, with global industry analysts expecting this value to rise to $430 billion by 2018 (M&A International INC. 2013).

**Table 1:** Global studies aimed at investigating the mislabelling rates of fish samples

Country	Number of samples analysed	Mislabelling rates (%)	References
Australia	38	0	Lamendin et al. (2015)
Brazil	30	24	Carvalho et al. (2015)
Canada	236	41	Hanner et al. (2011)
China	42	86	Xiong et al. (2016)
Egypt	90	33	Galal-Khallaf et al. (2014)
France	371	3.7	Bénard-Capelle et al. (2015)
Germany	145	6.2	Mariani et al. (2015)
India	100	22	Nagalakshmi et al. (2016)
Iran	27	11	Changizi et al. (2013)
Italy	69	32	Filonzi et al. (2010)
Japan	26	8	Viñas and Tudela (2009)
Malaysia	62	16	Chin et al. (2016)
Portugal	178	6.7	Mariani et al. (2015)
Republic of Ireland	131	28	Miller et al. (2012)
South Africa	149	18	Cawthorn et al. (2015)
Spain	245	7.8	Muñoz-Colmenero et al. (2016)
Turkey	50	86	Keskin and Atar (2012)
USA	216	13	Khaksar et al. (2015)
United Kingdom (UK)	386	5.7	Helyar et al. (2014)

Genomics, proteomics, metabolomics and lipidomics are four alternative and in some cases complimentary systems biological approaches often employed for food fraud detection studies (Ellis et al. 2016). The majority of fish fraud detection studies utilise genomic profiling as DNA is found in all cells and organisms and can be analysed in all types of tissue ranging from freshly caught fish to processed and cooked samples (Nielsen et al. 2012). Whilst very accurate qualitative and quantitative results are achievable using polymerase chain reaction (PCR), it comes at the expense of long and often complex sample preparations coupled with long assay running times. In terms of managing fraud in fast moving supply chains this is a substantial disadvantage.

Ambient mass spectrometry (AMS) is a relatively new field of analytical chemistry which is showing promise at detecting food fraud (Black et al. 2016). The recent increase in popularity of these techniques is a result of minimal or no sample preparation being required and fast assay running times. Whilst excellent qualitative results are achievable, it would appear quantitatively they struggle, especially with solid samples (Black et al. 2016; Hajslova et al. 2011). Whereas some food commodities such as meat (Montowska et al. 2015), dairy products (Hrbek et al. 2014), olive oil (Porcari et al. 2016) and spices (Shen et al. 2012) have been subjected to analysis using AMS techniques, fish has yet to receive the same level of investigation.

Rapid evaporative ionisation mass spectrometry (REIMS) is one of the newest forms of AMS and, as is the case with many analytical innovations was created for medical research purposes. It operates using an electrosurgical knife, bipolar forceps or laser which creates an aerosol (smoke) when cutting into a tissue sample. The aerosol is evacuated from the sample through a transfer line into the ionisation source of a mass spectrometer where a heated collision surface is situated and the ionisation process occurs. Although the majority of publications utilising REIMS have centred on medical (tissue identification) and bacterial identification applications (Balog et al. 2013; Strittmatter et al. 2014), there are early indications that it may also find applications in the detection of food fraud (Balog et al. 2016). Results are obtained near-instantaneously (2–3 s) and the technique appears to be able to achieve semi-quantitative results for solid samples without the need for any form of sample preparation within a liquid solution.

In the present study REIMS was applied to five commercially popular and genetically similar white fish species (cod, coley, haddock, pollock and whiting) and investigated as to whether fast and accurate speciation results could be obtained. The REIMS technology was believed to have the capability to determine the sample species in real time, unlike most forms of analytical systems employed for such studies. Additionally, this study demonstrates the possibility of distinguishing between different catch methods within a species, an aspect of fish fraud which is well known but has never been previously reported.

## Methods

### Sampling

This study was based upon five commercially popular white fish species. All tissue samples (fillets, tails and unspecified areas) of cod, coley, haddock, pollock and whiting were sourced from trusted suppliers and stored at − 80 °C. Samples of seabass and seabream fillets were sourced from Italy and stored at − 80 °C. Prior to REIMS analysis the samples were thawed at room temperature for 2 h in the fumehood where the REIMS cutting took place.

### REIMS experimental setup

The experimental setup for this study was similar to that reported previously (Balog et al. 2016). A Medimass REIMS source (Medimass, Budapest, Hungary) was mounted orthogonally to the interface of a Xevo G2-XS quadrupole time-of-flight (QTof) mass spectrometer (Waters Corporation., Wilmslow, UK) which was operated in negative ion and sensitivity mode. Mass spectra data were acquired over the range m/z 200–1200 with a scan time of 0.5 s. The REIMS source was connected to a monopolar electrosurgical knife (Model PS01-63H, Hangzhou medstar technology Co, Ltd, Jiaxing City, China) through a 3 m long, 1 cm. diameter ultra-flexible tubing (evacuation/vent line). Electrosurgical dissection in all experiments was performed using an Erbe VIO 50C generator (Erbe Medical UK Ltd, Leeds, UK). The generator was operated in ‘autocut’ mode with a power setting of 30W. All samples were cut on the return electrode and a venturi gas jet pump driven by nitrogen (1 bar) evacuated the aerosol produced at the sample site towards a heated kanthal coil that was operated at 6.4W (2.8 A @ 2.3 V). A lockmass solution of Leucine Enkephalin (LeuEnk) (m/z 554.2615) (2 ng/µL) in isopropanol (IPA) was infused using a Waters Acquity UPLC I-class system (Waters Corporation., Milford, MA, USA) at a continuous flow rate of 0.1 mL/min for accurate mass correction. Prior to analysis the mass spectrometer was calibrated using 5 mM sodium formate solution (90% IPA) at a flow rate of 0.2 mL/min for 2 min. Dependent on the size, each tissue sample was cut 8–12 times for reproducibility with each cut lasting approximately 3–5 s. This enabled multiple locations on each tissue sample to be analysed. The delay between sampling and appearance of a signal was ≈ 2 s, with no carry-over effects visible between each burn and/or sample.

### REIMS data pre-processing and analysis

Principal component analysis (PCA), an unsupervised technique, linear discriminant analysis (LDA) and orthogonal partial least squares-discriminant analysis (OPLS-DA), both supervised techniques, were used to build the qualitative speciation and catch method models within this study.

Raw data generated by the mass spectrometer were pre-processed using a prototype software (Waters Research Centre, Budapest, Hungary) that used standard Masslynx pre-processing algorithms (Waters). The recorded scans for each sample were combined to give an average spectrum and thus one spectrum for each sample was used to build the chemometric models. The resulting data were lockmass corrected using LeuEnk (m/z 554.2615) and normalised (Total Ion Count—TIC) before being exposed to multivariate analysis. All chemometric models were calculated using the mass region of m/z 600–950, a spectral intensity threshold of 2e^6^ counts and a bin width of 0.5 Da. When using a m/z range for models that included LeuEnk, variations in the lockmass intensity and interferences with the lockmass compound resulted in a degree of irreproducibility/error. PCA was used to reduce the dimensionality of the data prior to LDA analysis using the first 25 PCA components. The prototype software enabled a leave-20%-out cross-validation of the PCA-LDA score plots in which one average spectrum per sample was analysed. A model was calculated using 80% of the samples and data files left out were classified using the training model. This was repeated five times enabling each sample to be left out once from the model building process. Using a standard deviation of 5σ, each sample was classified to the closest class. If a sample was outside the standard deviation range of 5σ for all classes, then it was marked as an outlier.

The processed matrix generated within the prototype modelling software was exported to SIMCA 14 (Umetrics, Umea, Sweden) allowing the data to be exposed to further chemometric functions such as OPLS-DA. All data was mean-centered, pareto scaled and grouped accordingly into the five species of fish. R^2^ (cumulative), Q^2^ (cumulative) and a misclassification table were used to determine the validity of the models. R^2^ (cum) indicates the variation described by all components in the model and Q^2^ (cum) is a measure of how accurately the model can predict class membership. Permutation tests were carried out to ensure the models were not over-fitted. Individual OPLS-DA speciation models and S-plots of each species of fish against the other four species were generated to identify ions of significance for each species.

### Real time recognition of samples

The PCA-LDA models created using the prototype software were exported to a prototype recognition software (Waters Research Centre, Budapest, Hungary) allowing for real-time identification of samples. Raw data files were acquired and ran live though the software providing a near-instantaneous identification, excluding the delay between sampling and appearance of a signal which was ≈ 2 s. A standard deviation of 5σ was used for class assignment. The spectral intensity limit was set at 1e^8^ counts thus ensuring that only the cuts were assigned a species classification and not any background noise.

### DNA analysis setup and analysis

Mitochondrial cytochrome c oxidase subunit I gene (*COI*) was used as genetic marker for the examination of samples. DNA extraction was performed using a commercial kit (NucleoSpin Tissue—Macherey Nagel) according to the manufacturer guidelines. A fragment of approximately 655 bp of *COI* was amplified using the primer pair COIfish_F1 (5′-TCAACYAATCAYAAAGATATYGGCAC-3′) and COIfish_R1 (5′-ACTTCYGGGTGRCCRAARAATCA-3′) in a PCR reaction (Ward et al. 2005). The sequences were determined by direct DNA sequencing on both strands of the PCR products by BigDye Terminator v3.1 cycle sequencing kit using the amplification primer pair and analysed on ABI Prism 3130 Genetic Analyzer (Applied Biosystems). Sequences were compared with those deposited in GenBank and Barcode of Life Data Systems (BOLD). Results were considered valid above 98% of similarity.

## Results

### REIMS fish speciation

**Fig. 1:**
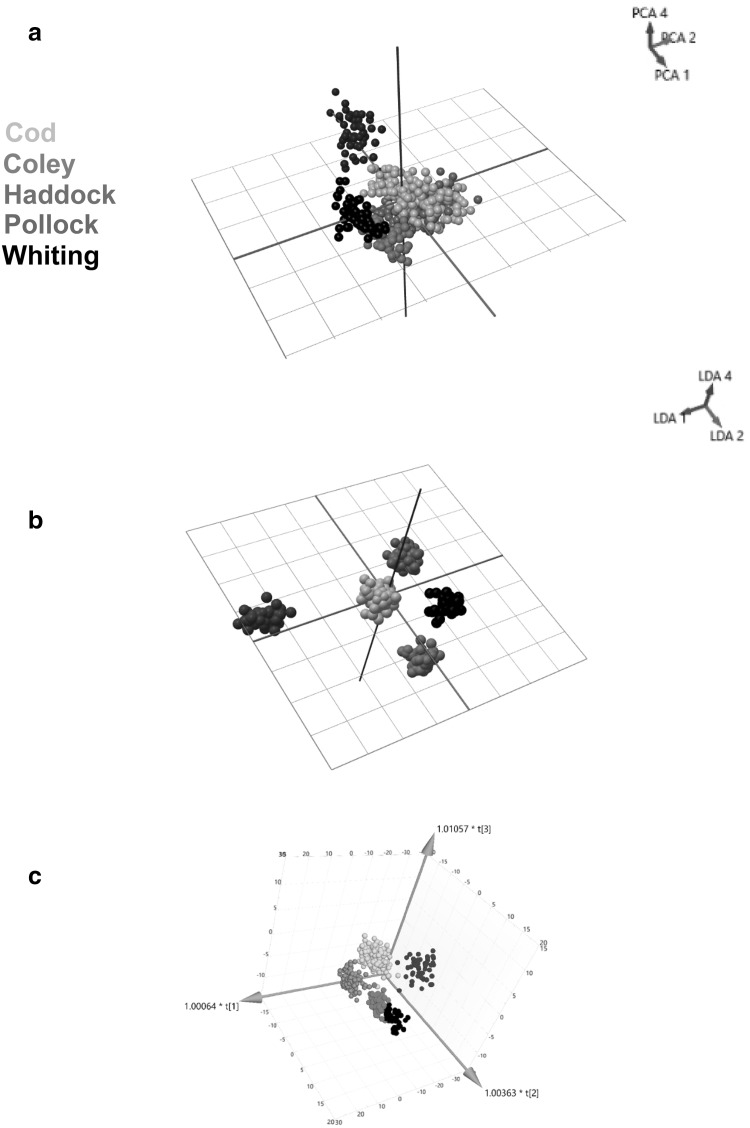
**a** Principal component analysis (PCA), **b** linear discriminant analysis (LDA) and **c** orthogonal partial least squares-discriminant analysis (OPLS-DA) models generated using the prototype software and SIMCA 14. All models were generated using the mass range m/z 600–950 of the fish samples with clear separation of the five fish species of fish; cod (orange), coley (red), haddock (green), pollock (blue) and whiting (black) visible within the 3-D LDA and OPLS-DA models

Raw spectrometric data (Supplementary Information S1) obtained from authenticated samples of cod (n = 194), coley (n = 51), haddock (n = 133), pollock (n = 50) and whiting (n = 50) were pre-processed and subjected to multivariate analysis where PCA, LDA and OPLS-DA were applied. 80 PCA components and 4 LDA components were used to generate the chemometric models. Clustering was identified within the three-dimensional (3-D) PCA score plot using components 1, 2 and 4 (Fig. 1a). However, clear separation between the five species of fish was obtained within the 3-D LDA score plot using components 1,2 and 4 (Fig. 1b) and the OPLS-DA score plot where 4 latent and 4 orthogonal components were used (Fig. 1c). A leave-20%-out cross-validation of the PCA-LDA models, where one average spectrum per sample was used resulted in a 99.37% correct classification (Supplementary Information S2) which was due to two samples being assigned an outlier classification and one whiting being identified as coley. Additionally, a correct classification rate of 99.37% was obtained for the OPLS-DA model (Supplementary Information S3) which was due to two cod samples being identified as coley and whiting, and one coley sample being identified as whiting. R^2^ and Q^2^ values of 0.829 and 0.809 indicated that the OPLS-DA model had both a good quality of fit and predictivity towards new data. A large Q^2^ value also suggests that the multivariate data points are well clustered with there being very few outliers within the dataset as exemplified in all the chemometric models within Fig. 1. The relevant permutation tests (Supplementary Information S4) were carried out to demonstrate that the models were not over-fitted.

### Real time validation of speciation model

**Fig. 2:**
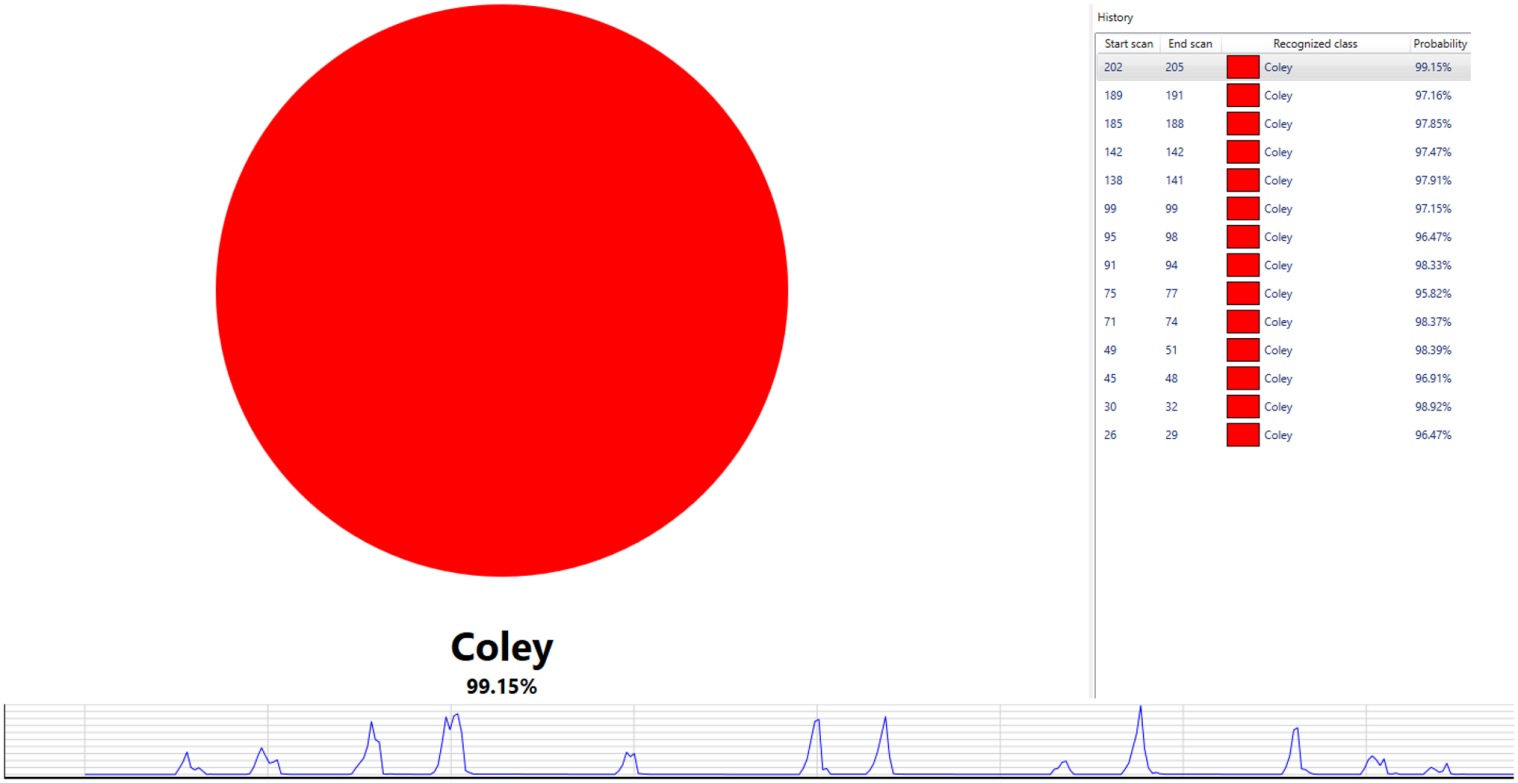
Validation of the speciation models using the prototype recognition software and a further set of authenticated fish samples. In this scenario, the sample under investigation is coley and the figure above demonstrates the recognition software correctly identifying a sample burn to be coley (red circle). The results for each burn are obtained near-instantaneously excluding the delay between sampling and appearance of a signal which was ≈ 2 s. Twelve cuts were taken from this sample which is identified in the chromatogram with identification for some of the cuts identified on the right-hand side of the figure. A standard deviation of 5σ was used for class assignment. Of the 99 samples analysed, 98 (98.99%) were correctly identified with one cod sample being assigned as an outlier

Raw spectrometric data obtained from authenticated samples of cod (n = 22), coley (n = 20), haddock (n = 20), pollock (n = 20) and whiting (n = 17), which had not been previously used to generate the chemometric models were run live through the prototype recognition software providing a near-instantaneous (≈ 2 s) identification (Fig. 2). Of the 99 samples analysed, 98 (98.99%) were correctly identified with one cod sample being assigned as an outlier (unidentified).

### Statistical validation of speciation model

The second approach of the validation was carried out to ensure the validity of the results from the prototype recognition software. The raw data acquired from the 99 samples were subjected to a cross-validation similar to that of the leave-20%-out cross-validation. A model was created using the training set of samples used to generate the speciation models (n = 478) excluding the 99 validation samples. Each validation sample was then assigned a fish species classification using one average spectrum and a standard deviation of 5σ. The results were in agreement to that of the recognition software and a correct classification rate of 98.99% was obtained (Supplementary Information S5).

### DNA analysis of suspect ‘haddock’ samples

During the investigation and generation of the speciation models it was found that six samples labelled as ‘haddock’ were clustered within the cod samples in all chemometric models. Additionally, the prototype recognition software identified all six ‘haddock’ samples as cod in which it took 15/20 min to obtain results for all the samples. As a result, the samples were further analysed using PCR to establish whether they were indeed haddock or whether they had accidentally been mislabelled. Mitochondrial cytochrome c oxidase subunit I gene (*COI*) was used as genetic marker for the six samples, in which all showed 99% similarity with *Gadus morhua* species (cod) on both Genbank and BOLD. No significant similarities were observed with *Melanogrammus aeglefinus* (haddock).

### Real time analysis of seabass and seabream samples

Raw spectrometric data obtained from authenticated samples of seabass (n = 6) and seabream (n = 8) were simultaneously run live through the prototype recognition software providing a near-instantaneous (≈ 2 s) classification. Of the 14 samples analysed, 13 (92.86%) were correctly identified as outliers with one sample being identified as both an outlier (66%) and coley (34%) sample.

### Statistical validation of seabass and seabream samples

The second approach of the validation was carried out to ensure the validity of the results from the prototype recognition software. The raw data acquired from the 14 samples were subjected to a cross-validation like that of the leave-20%-out cross-validation. A model was created using the training set of samples used to generate the speciation models (n = 478) excluding the 14 seabass and seabream samples. Each sample was then assigned a fish species classification using one average spectrum and a standard deviation of 5σ. An overall correct classification rate of 100% for all 14 samples was obtained as the cross-validation uses a single averaged spectrum of all the cuts per sample resulting in the one seabream sample which was assigned as both an outlier (66%) and coley (34%) sample being assigned an outlier classification.

### Catch method of haddock

**Fig. 3:**
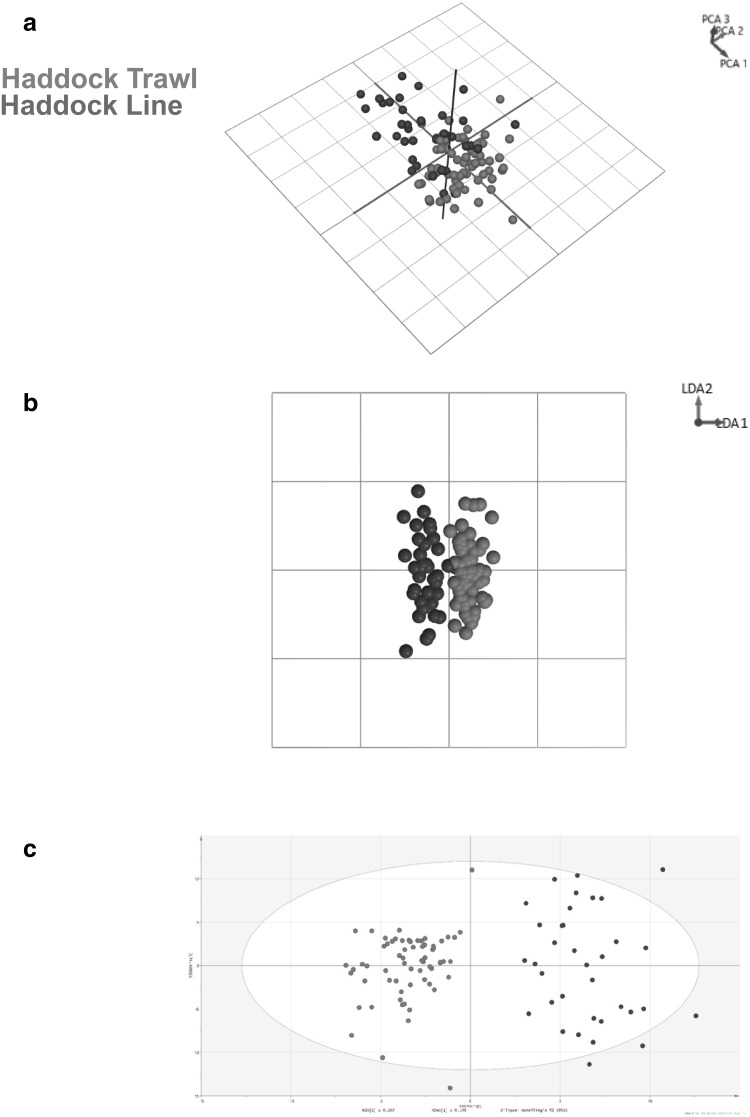
**a** Principal component analysis (PCA), **b** linear discriminant analysis (LDA) and **c** orthogonal partial least squares-discriminant analysis (OPLS-DA) models generated using the prototype software and SIMCA 14. All models were generated using the mass range m/z 600–950 of the fish samples with clear separation of the two catch methods; haddock trawl (red) and haddock line (blue) evident within the 2-D LDA and 3-D OPLS-DA models

Raw spectrometric data obtained from both line caught (n = 35) and trawl caught (n = 65) haddock samples were exposed to multivariate analysis allowing PCA, LDA and OPLS-DA models to be generated. 20 PCA components and 2 LDA components were used to generate the catch method models. Some separation was apparent within the 3-D PCA score plot using components 1, 2 and 3 (Fig. 3a). However, clear separation was attained in the two-dimensional (2-D) LDA score plot using components 1 and 2 (Fig. 3b), and the OPLS-DA score plot (Fig. 3c) in which 1 latent and 3 orthogonal components were used. A leave-20%-out cross-validation of the PCA-LDA models resulted in a 95.00% correct classification with three trawl caught and two line caught samples being misidentified (Supplementary Information S6). However, a correct classification rate of 100% was obtained for the OPLS-DA model. R^2^ and Q^2^ values of 0.863 and 0.746 were obtained suggesting that the OPLS-DA model was both robust and had good predictability towards a new set of data. The relevant permutation tests (Supplementary Information S7) were carried out to demonstrate that the models were not over-fitted.

## Discussion

Industries across the food sector want fast and accurate results when undertaking their own quality control (QC) checks. DNA approaches, of which most of the studies in Table 1 have employed, fulfil the criteria of obtaining accurate results, but it comes at the expense of long sample preparations and assay running times. Validation of the chemometric speciation models, in which a 98.99% correct classification (Table 2) was achieved using the prototype recognition software (Fig. 2) clearly shows that REIMS can fulfil the principle of real time profiling without sacrificing the quality of results that are obtained. Considering that no sample preparation is required, which is a major pitfall for PCR, it is evident that REIMS and maybe other AMS techniques (Porcari et al. 2012) have a prominent role to play in tackling fish fraud. As each sample is cut 8–12 times it could be possible that the raw data acquired using REIMS is analogous to that of liquid chromatography-mass spectrometry (LC-MS), a ‘classical’ technique often used when carrying out metabolomic profiling experiments. Perhaps from an analytical variability standpoint (QC pooled samples) LC-MS is more suited towards such metabolomic profiling experiments (De Vos et al. 2007). But, in a real-world situation where species identification is both desired and needed rapidly (fishery, port loading dock, etc.) LC-MS cannot compete with the REIMS technology.

**Table 2:** Putative identifications of the three pollock ions identified in Fig. 4 and the ion found to be most significant for the separation of the other four species of fish in the chemometric models

Species	m/z (Da)	Collision energy (V)	Ion	Lipid class	Fragment (s) (m/z–Da)	Putative identification
Cod	788.5	30	[M-H]^−^	PE	327.24	22:6/18:1
					281.25	
					153.00	
				PS	283.26	18:1/18:0
					281.25	
					153.00	
				PS	309.28	20:1/16:0
					255.23	
					153.00	
Coley	817.5	35	N/A	N/A	327.24	N/A
					283.25	
					281.25	
					255.23	
					229.20	
Haddock	810.5	35	[M-H]^−^	PE	327.24	22:6/20:4
					303.24	
					283.25	
					153.00	
				PE	301.22	22:5/20:5
					257.23	
					153.00	
				PS	303.24	20:4/18:0
					283.25	
					153.00	
Pollock	629.5	20	N/A	N/A	327.24	N/A
					301.22	
					283.25	
	655.5	15	[2M-H]^−^	FA	327.24	22:6
					283.25	
					229.20	
	667.5	25	N/A	N/A	339.21	N/A
					327.24	
					301.22	
					283.25	
					257.24	
Whiting	790.5	30	[M-H]^−^	PE	327.24	22:6/18:0
					283.25	
				PS	283.25	18:0/18:0
			N/A	N/A	701.42	N/A
					480.33	
					463.24	
					255.25	

Two different classes of phospholipids; phosphatidylethanolamine (PE) and phosphatidylserine (PS) were found to be the most likely identification for the ions with the only exception being the pollock ion m/z 655.5 which is believed to be a dimer of docosahexaenoic acid (DHA) (m/z 327.21 [M-H]^−^)

**Fig. 4:**
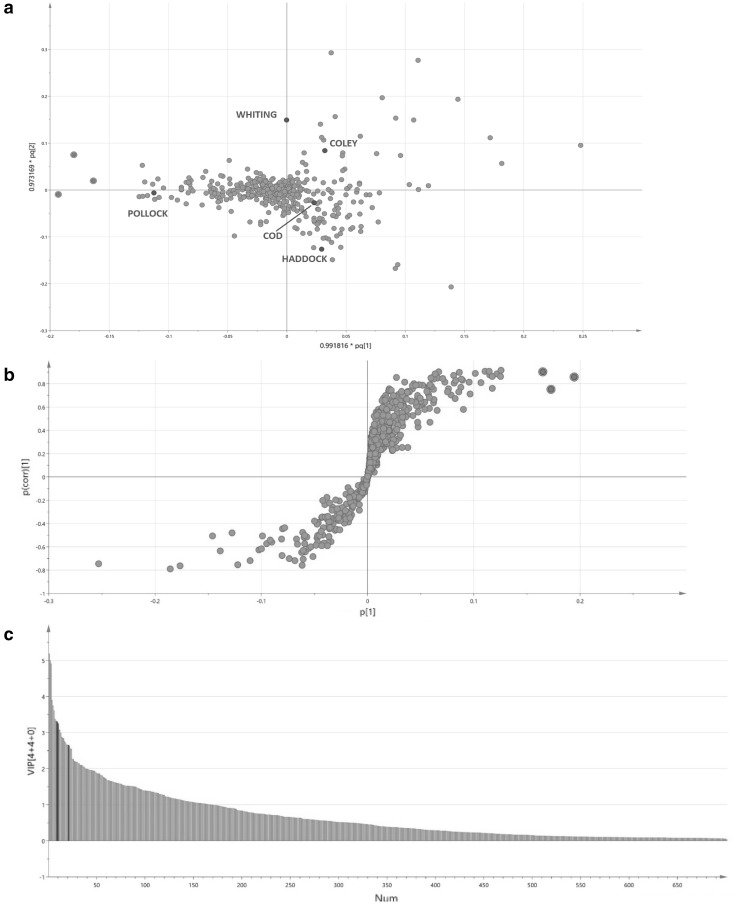
Method to identify ions which are found predominately within pollock compared to that of the other four species of fish; **a** a PCA loading plot identifying the average position of each species of fish (blue markers) and the relevant ions (green markers) that contribute most to their positioning; **b** a S-plot of pollock v the other species of fish identifying the ions that are found predominately in pollock; **c** a VIP graph of all 701 ions analysed in the multivariate dataset. The three ions identified within the loading and S-plots (red) have great significance (VIP > 1, S-plot |p| > 0.03 and S-plot |p(corr)| > 0.5) towards the dataset and explain the separation of pollock from the other four species of fish within the PCA score plot. Based on MS/MS fragmentation, two of the three ions (m/z 629.5 and 667.5) could not be assigned a putative identification. However, m/z 655.5 [2M-H]^−^ was identified as a dimer of docosahexaenoic acid (DHA) (m/z 327.21 [M-H]^−^). Table 2 identifies all fragment ions

The mislabelling of the six ‘haddock’ samples signifies the vast time comparisons that exist between PCR and REIMS. Whereas the REIMS technology in conjunction with the prototype recognition software provided a result for each sample burn within seconds (including sample preparation), PCR analysis of the six samples took 24 h, including time taken for sample preparation. Both analytical platforms produced identical results and it is evident that REIMS has the capability to analyse many samples within the timeframe taken for a PCR result. These time-based comparisons are very significant as it demonstrates how companies with fast moving supply chains could be operating their own QC checks in the future, with fast and accurate results attainable within seconds which is ultimately what they desire.

Fast results are coveted but not at the expense of false positive and negative identifications. The versatility of the REIMS and strength of the chemometric models, evaluated by R^2^ = 0.829, Q^2^ = 0.809 and the permutation tests (Supplementary Information S4), is also demonstrated by the eight seabream and six seabass samples. All 14 samples were correctly identified as outliers with one seabream sample being assigned both an outlier (66%) and coley (34%) sample. However, because a greater majority of the cuts were identified as an outlier and not coley, the statistical validation of all 14 samples gave a 100% correct classification as the software uses one average spectrum of all the cuts for each sample. Along with the validation of the speciation models and the PCR testing of the six suspect ‘haddock’ samples, the classification of the 14 seabass and seabream samples as outliers further illustrates that fish speciation is very achievable using REIMS with fast and accurate results attainable. Compared to PCR, the coupling of the REIMS source to a XEVO G2-XS QTof mass spectrometer does result in large cost differences. However, in this study only a few aspects of the QTof mass spectrometer were utilised; the time-of-flight (Tof) tube and the detector. MS/MS functions such as the quadrupole and collision induced dissociation (CID) were not and therefore, it may be possible to couple the REIMS source to a cheaper and perhaps smaller alternative as the development of miniaturised and fieldable mass spectrometers appears to be making significant advances (Snyder et al. 2016). Paper spray (PS), desorption atmospheric pressure chemical ionisation (DAPCI) and several other AMS plasma based sources [dielectric barrier discharge ionisation (DBDI), low temperature plasma ionisation (LTP) and plasma-assisted desorption ionisation (PADI)] have reportedly been coupled to a miniature mass spectrometer instrument (Snyder et al. 2016). However, in practice it will be a long time until the use of miniaturised mass spectrometers becomes common practice.

The aim of this study was to demonstrate that REIMS can be used as a fast profiling technique which the fish and perhaps the whole food industry can use to carry out QC checks and that there are significant time comparisons that exist between REIMS and techniques that are commonly associated with such studies like PCR and LC-MS. Yet, within the study it has been found that there are potential ions of significance for pollock (Fig. 4a–c) and the other four species of fish (Supplementary Information S8, S9). The significance of the chosen ions was exhibited by their variable importance in projection (VIP) values (x > 1), their S-plot |p| values (x > 0.03) and their S-plot |p(corr)| values (x > 0.5). Putative identifications were assigned by carrying out a targeted MS/MS approach which involved collision induced dissociation (CID) to obtain fragments for the three pollock ions identified in Fig. 4 and the ion thought to have the greatest influence towards the separation of the other four species of fish within the chemometric models. Based on previous studies carried out using the REIMS technology and the mass range that we have utilised to generate the chemometric models, we expected the ions to be phospholipids (Balog et al. 2013; Verplanken et al. 2017). Putative identifications could not be assigned to every ion but the fragments identified in Table 2 suggest a mixture of isobaric and isomeric phospholipid species and/or the presence of other lipid species. For the ions of which it was possible to assign a classification, it is believed that they are most likely to be one of two different classes of phospholipid; phosphatidylethanolamine (PE) and phosphatidylserine (PS). Multiple lipid classes have been assigned due to the lack of chromatographic separation that accompanies REIMS analysis. The only ion not to be identified as a phospholipid species was m/z 655.5 [2M-H]^−^ which is believed to be a dimer of docosahexaenoic acid (DHA) (m/z 327.21 [M-H]^−^). Fragment ions of m/z 283.25 suggest loss of CO_2_ from DHA and m/z 229.20 suggests a McLafferty rearrangement.

Substitution of one species of fish for another is by far the most commonly reported with regards to fish fraud. However, there are six other forms in which it can manifest itself; IUU fishing; fishery substitution; processed raw material authenticity (species adulteration); chain of custody abuse; undeclared product content and catch method (Elliot 2014). To date, the scientific investigation of different catch methods within the same species of fish has never been reported. Separation of the two haddock catch methods was achieved (Fig. 3a–c) but it is unclear as to whether this was due to genuine differences in which way the fish samples were caught. REIMS spectral data are thought to be dominated by intact phospholipids and fatty acids. However, differences in the catch method of a fish would not be thought to affect the lipid profile of a fish unless they had different diets which may be a result of line caught fish being caught at shallower depths compared to that of trawl caught samples. A more plausible explanation is that the two different catch methods are likely to affect secondary metabolites (stress markers) within a fish sample. Compared to speciation, multivariate analysis of the catch method data did not result in any reliable ions that could explain separation within the models. The two ions believed to provide the greatest variance between the two catch methods, according to the S-plot, were m/z 764.5 and m/z 819.5 with the former thought to occur at more abundant levels in trawl caught samples and the latter in line caught samples. Similar to the speciation results, it is expected that numerous isobaric and isomeric lipid species are assignable to the two masses due to the lack of chromatographic separation that occurs within REIMS analysis. A search of known stress markers did not result in any assignments either. A larger study with equal amounts of samples for each class is required to confirm this. However, whichever whichever scenario it may be, separation between the two catch methods has been achieved and therefore, this is the first scientific study to demonstrate that differentiating between line and trawl caught samples within the same species is possible.

## Conclusions

No sample preparation, accurate and near-instantaneous results are three properties which the REIMS technology has exemplified in this study and are all three issues which cannot be fulfilled by most analytical platforms used for such fish studies. The large time comparisons (15/20 min–24 h) observed between REIMS and PCR to determine the species of six mislabelled samples are hugely significant. REIMS is a frontier technology not found in common analytical laboratories but it is clear that it has the potential to be utilised in commercial environments. In the short run it could be seen as a complimentary, albeit expensive technique to help aid the detection of commercial fish fraud whilst in the long run a miniaturised and cheaper version of the technology could be utilised by fisheries to conduct their own QC checks. As well as this, REIMS has shown to be able to analyse multiple aspects of fish fraud through the separation of line and trawl caught haddock samples and it may well be that there are other aspects such as geographic origin and wild/farmed which can be differentiated, further issues which genomic profiling is ill-equipped to do.

## Electronic supplementary material

Below is the link to the electronic supplementary material.

Supplementary material 1 (DOCX 2521 KB)
