# Supplementary material for: A real time metabolomic profiling approach to detecting fish fraud using rapid evaporative ionisation mass spectrometry
Source: Metabolomics. 2017 Nov 2;13(12):153. doi: 10.1007/s11306-017-1291-y (PMC5668337; doi:10.1007/s11306-017-1291-y)
Supplement: Supplementary file 1 — Supplementary material 1 (DOCX 2521 KB) [file 11306_2017_1291_MOESM1_ESM.docx]

**WHITING**

**POLLOCK**

**COD**

**COLEY**

**HADDOCK**

S1 – Raw background subtracted data (m/z 600-950) obtained from cod, coley, haddock, pollock and whiting fish samples acquired using REIMS operated in negative ion and sensitivity mode.

|  | **Cod** | **Coley** | **Haddock** | **Pollock** | **Whiting** | **Outlier** | **Total** | **Correct classification rate (%)** |
| --- | --- | --- | --- | --- | --- | --- | --- | --- |
| **Cod** | 192 | 1 | 0 | 0 | 1 | 0 | 194 | 98.97 |
| **Coley** | 0 | 51 | 0 | 0 | 0 | 0 | 51 | 100.00 |
| **Haddock** | 0 | 0 | 133 | 0 | 0 | 0 | 133 | 100.00 |
| **Pollock** | 0 | 0 | 0 | 50 | 0 | 0 | 50 | 100.00 |
| **Whiting** | 0 | 1 | 0 | 0 | 49 | 0 | 50 | 98.00 |
| **Total** |  |  |  |  |  |  | 478 | 99.37 |

|  | **Cod** | **Coley** | **Haddock** | **Pollock** | **Whiting** | **Outlier** | **Total** | **Correct classification rate (%)** |
| --- | --- | --- | --- | --- | --- | --- | --- | --- |
| **Cod** | 193 | 0 | 0 | 0 | 0 | 1 | 194 | 99.48 |
| **Coley** | 0 | 51 | 0 | 0 | 0 | 0 | 51 | 100.00 |
| **Haddock** | 0 | 0 | 132 | 0 | 0 | 1 | 133 | 99.25 |
| **Pollock** | 0 | 0 | 0 | 50 | 0 | 0 | 50 | 100.00 |
| **Whiting** | 0 | 1 | 0 | 0 | 49 | 0 | 50 | 98.00 |
| **Total** |  |  |  |  |  |  | 478 | 99.37 |

S2 - Results from the leave-20%-out cross-validation of the PCA-LDA speciation models generated using the prototype software in which an overall correct classification rate of 99.37% was achieved. Of the 478 samples analysed, only 3 were not assigned the correct species classification.

S3 - Results from the misidentification table of the speciation OPLS-DA model generated using SIMCA 14 in which an overall correct classification rate of 99.37% was achieved. Of the 478 samples analysed, only 3 were not assigned the correct species classification.


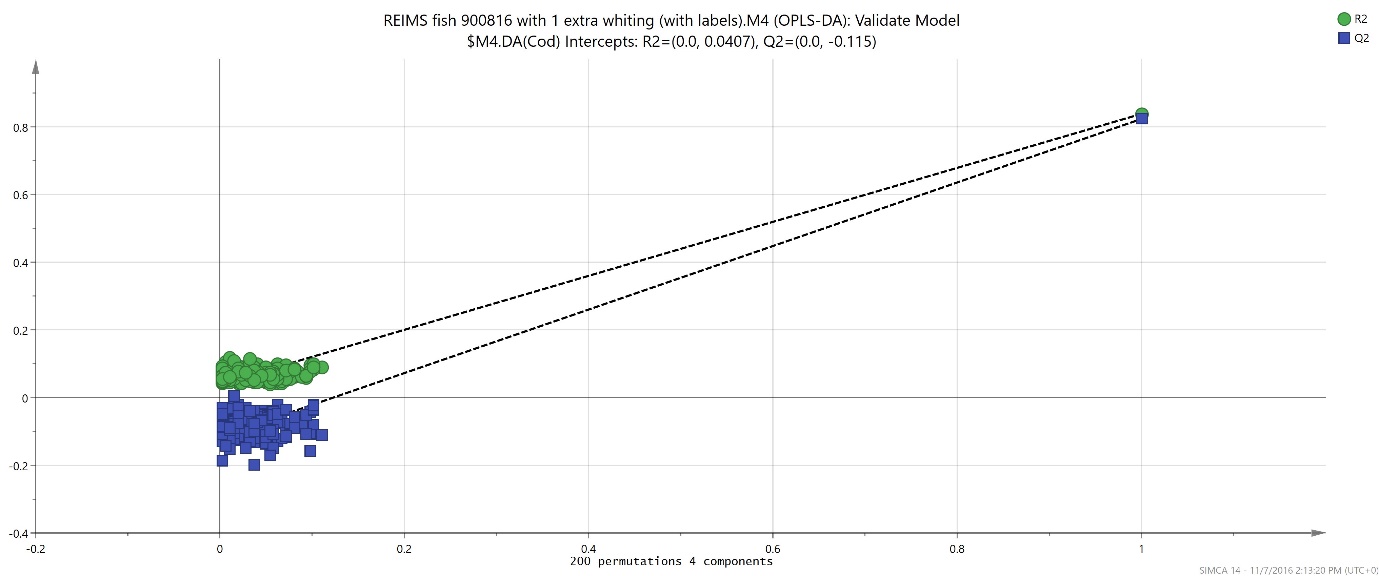


(a)


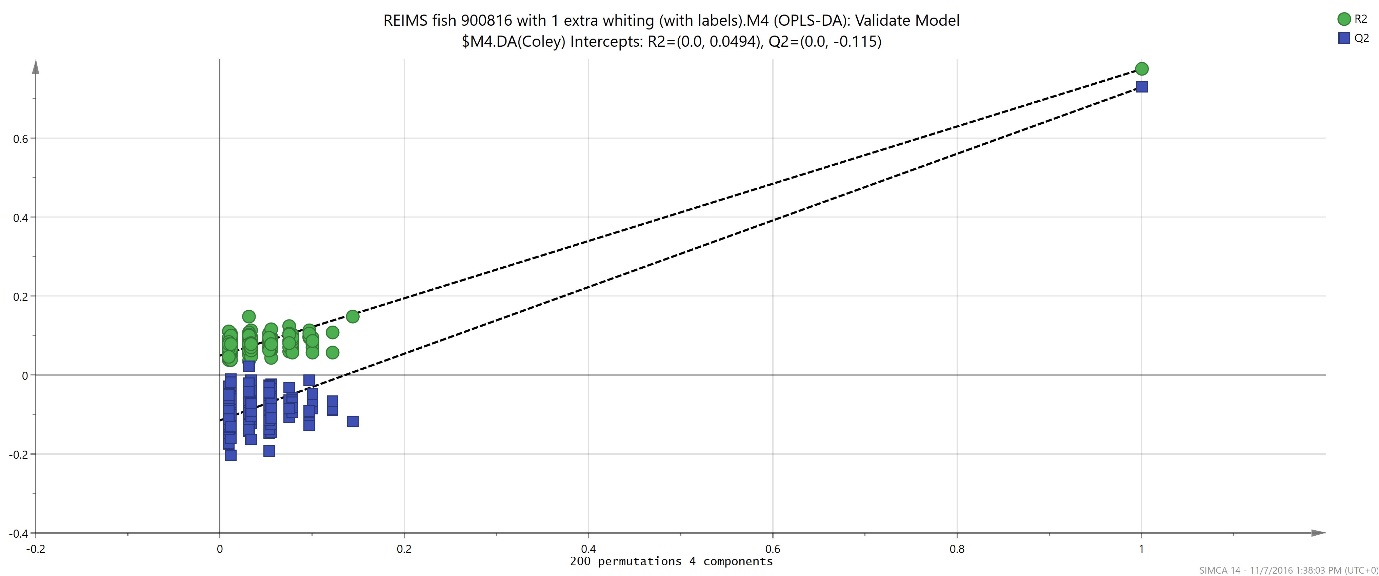


(b)

(c)


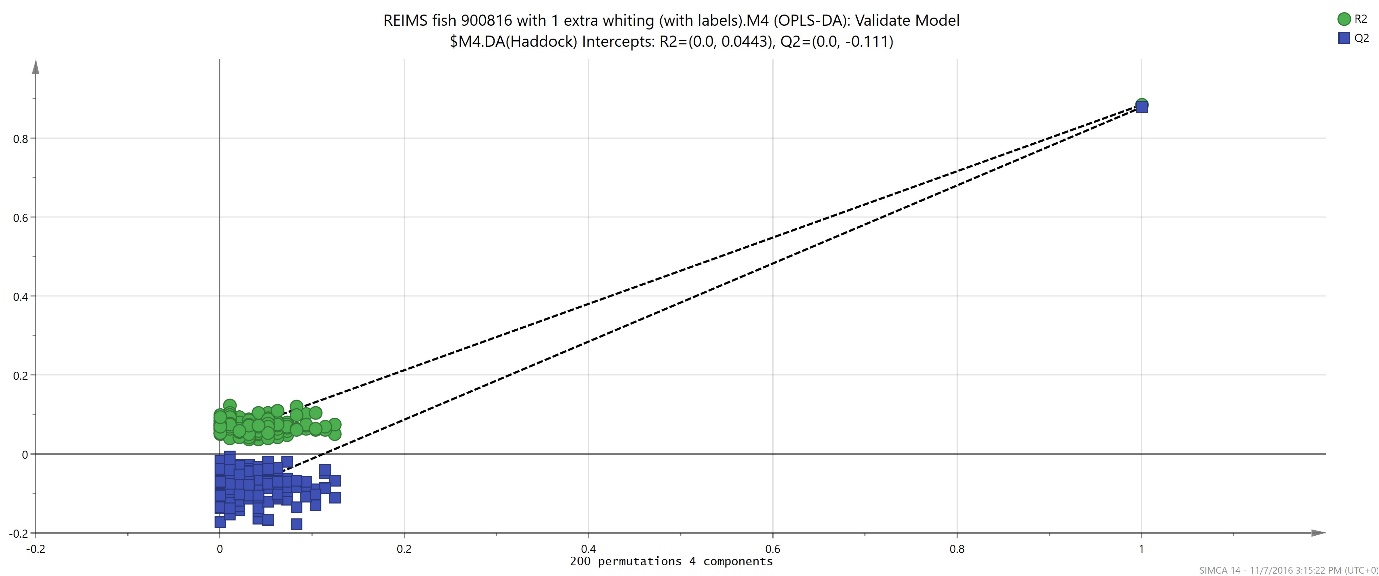


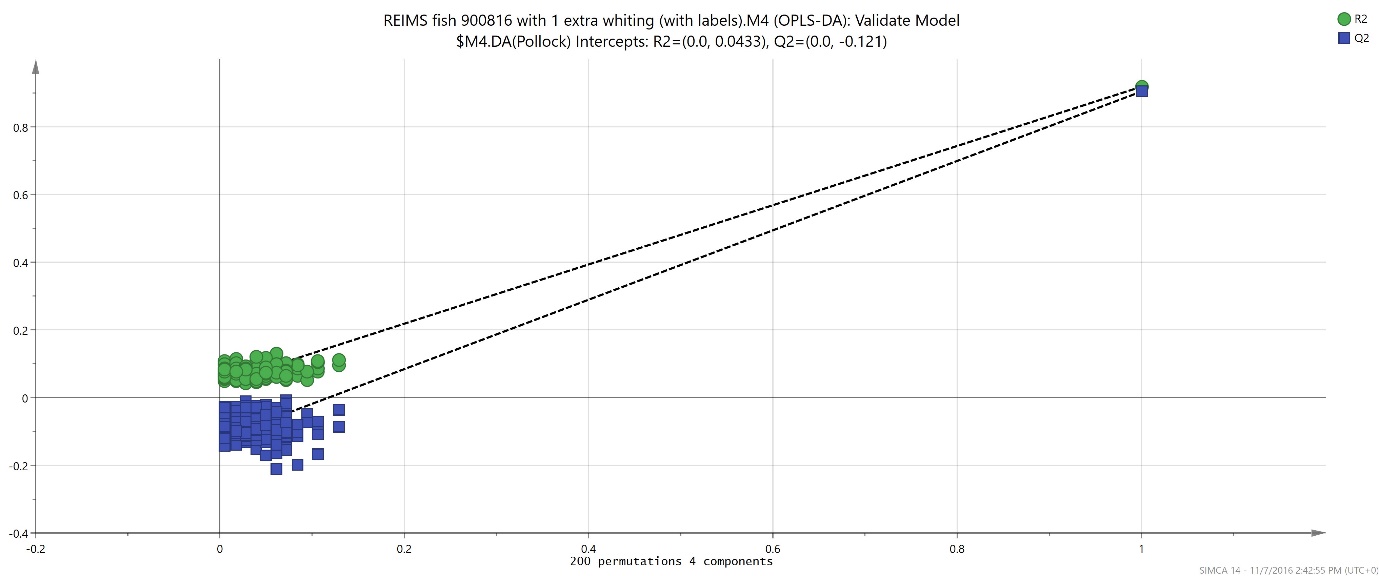


(d)


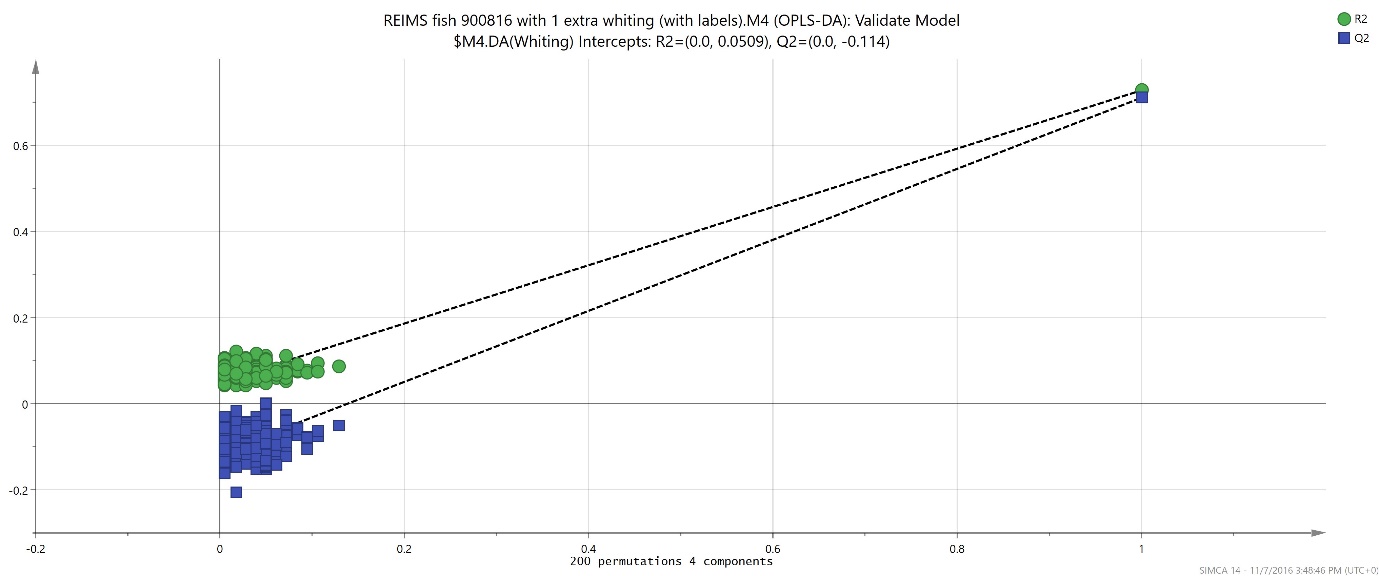


(e)

S4- Permutation tests for each species of fish within the OPLS-DA model (figure 1c); (a) cod; (b) coley; (c) haddock; (d) pollock and (e) whiting. All tests were carried out using 200 permutations with each Q^2^ regression line (blue) intercepting the y-axis beneath the origin suggesting that the OPLS-DA model was not over fitted.

|  | **Cod** | **Coley** | **Haddock** | **Pollock** | **Whiting** | **Outlier** | **Total** | **Correct classification rate (%)** |
| --- | --- | --- | --- | --- | --- | --- | --- | --- |
| **Cod** | 21 | 0 | 0 | 0 | 0 | 1 | 22 | 95.45 |
| **Coley** | 0 | 20 | 0 | 0 | 0 | 0 | 20 | 100.00 |
| **Haddock** | 0 | 0 | 20 | 0 | 0 | 0 | 20 | 100.00 |
| **Pollock** | 0 | 0 | 0 | 20 | 0 | 0 | 20 | 100.00 |
| **Whiting** | 0 | 0 | 0 | 0 | 17 | 0 | 17 | 100.00 |
| **Total** |  |  |  |  |  |  | 99 | 98.99 |

S5 - Statistical validation of the speciation models using the prototype software cross validation ensuring the results from the prototype recognition software were accurate. All samples were assigned the correct fish species except one cod sample which was identified as an outlier resulting in an overall correct classification rate of 98.99%.

|  | **Haddock Line** | **Haddock Trawl** | **Total** | **Correct classification rate (%)** |
| --- | --- | --- | --- | --- |
| **Haddock Line** | 33 | 2 | 35 | 94.29 |
| **Haddock Trawl** | 3 | 62 | 65 | 95.38 |
| **Total** |  |  | 100 | 95.00 |

S6 - Results from the misidentification table of the catch method OPLS-DA model generated using SIMCA 14 in which a 100% correct classification was obtained.


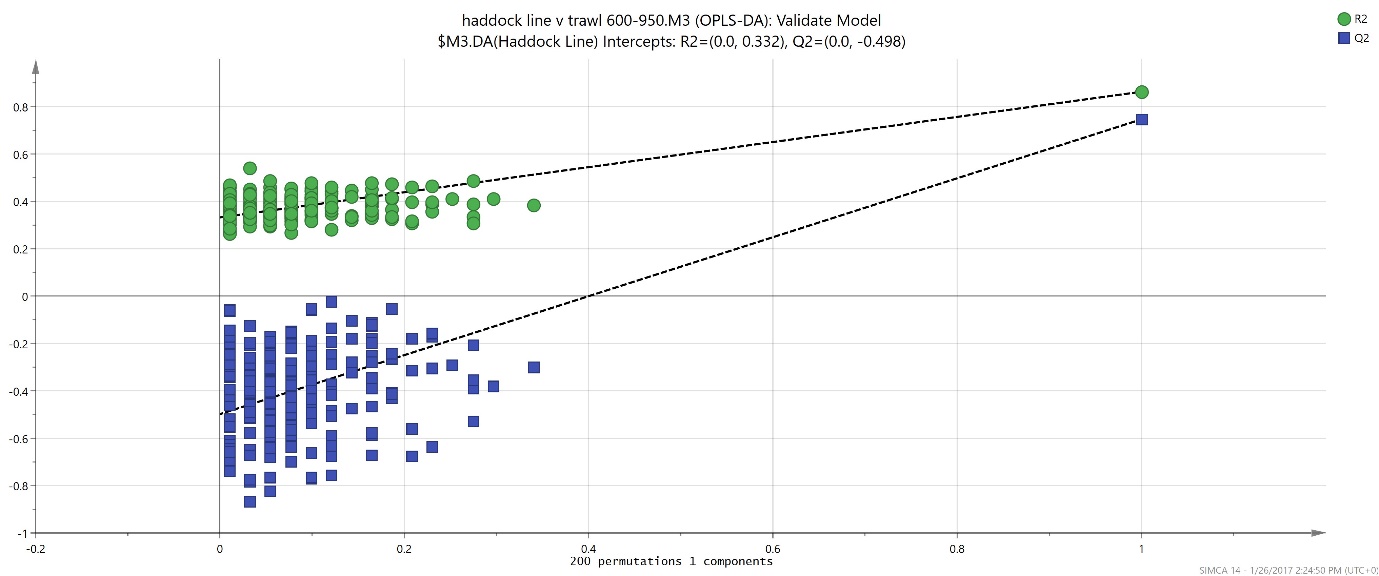


(a)


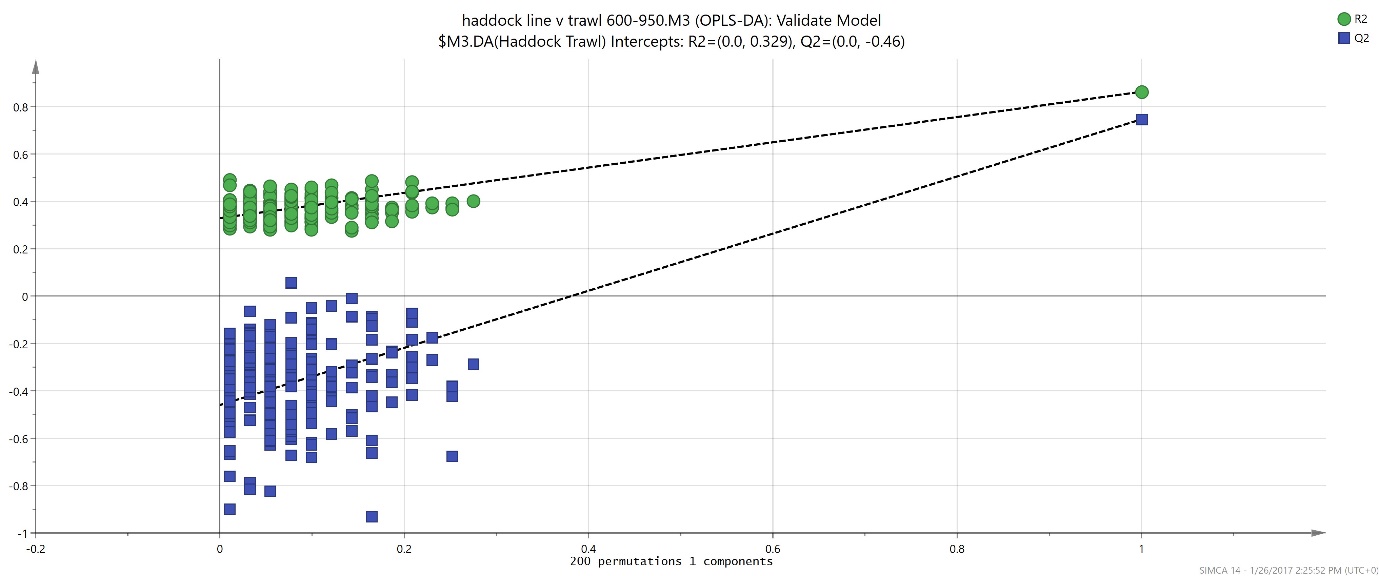


(b)

S7 - Permutation tests for each catch method of haddock within the OPLS-DA model (figure 3c); (a) haddock line and (b) haddock trawl. All tests were carried out using 200 permutations with each Q^2^ regression line (blue) intercepting the y-axis beneath the origin suggesting that the OPLS-DA model was not over fitted.

| OPLS-DA model | Latent component | Orthogonal component | R^2^ (cum) | Q^2^ (cum) | RMSECV |
| --- | --- | --- | --- | --- | --- |
| Cod v other species | 1 | 5 | 0.903 | 0.886 | 0.166 |
| Coley v other species | 1 | 9 | 0.909 | 0.857 | 0.117 |
| Haddock v other species | 1 | 4 | 0.930 | 0.923 | 0.124 |
| Pollock v other species | 1 | 5 | 0.954 | 0.932 | 0.080 |
| Whiting v other species | 1 | 6 | 0.874 | 0.829 | 0.127 |

S8- Values of the statistical parameters obtained for the different OPLS-DA models of each species of fish against the other four species under investigation generated using REIMS in negative ionisation mode to identify ions of significance.


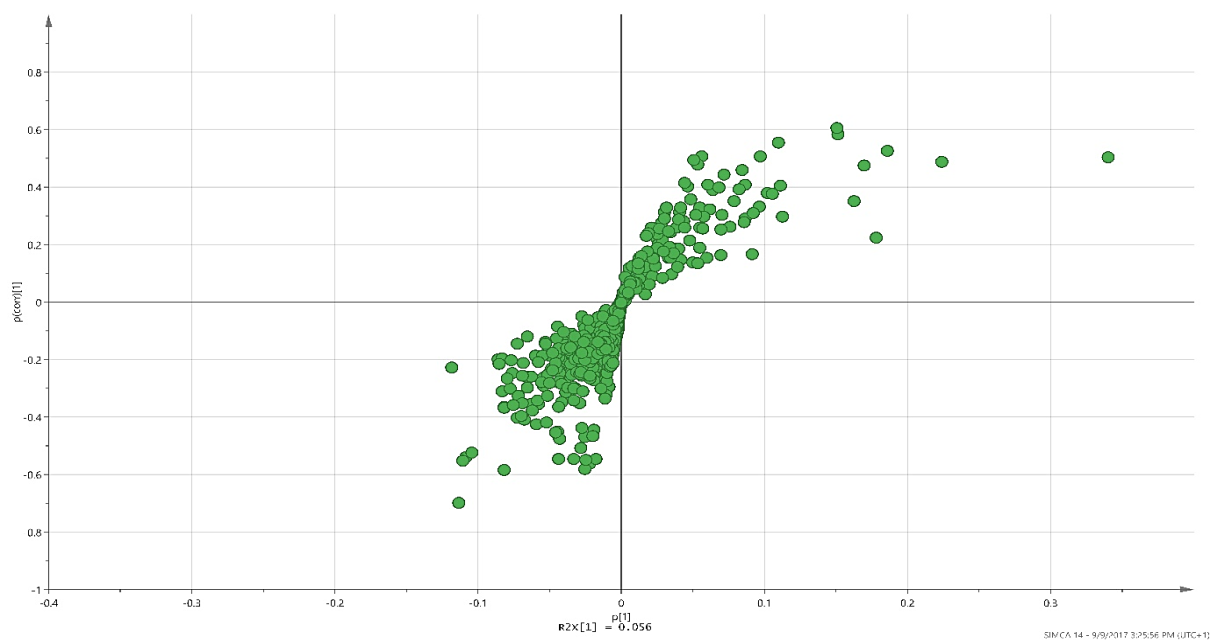


(a)

(b)


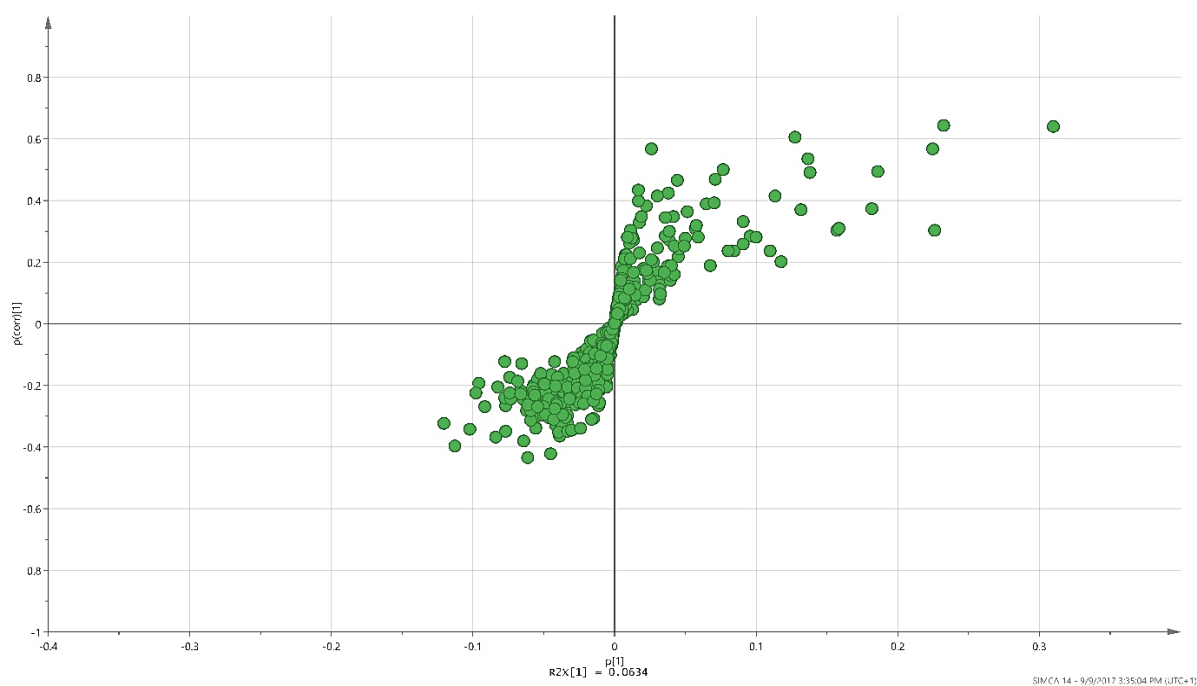


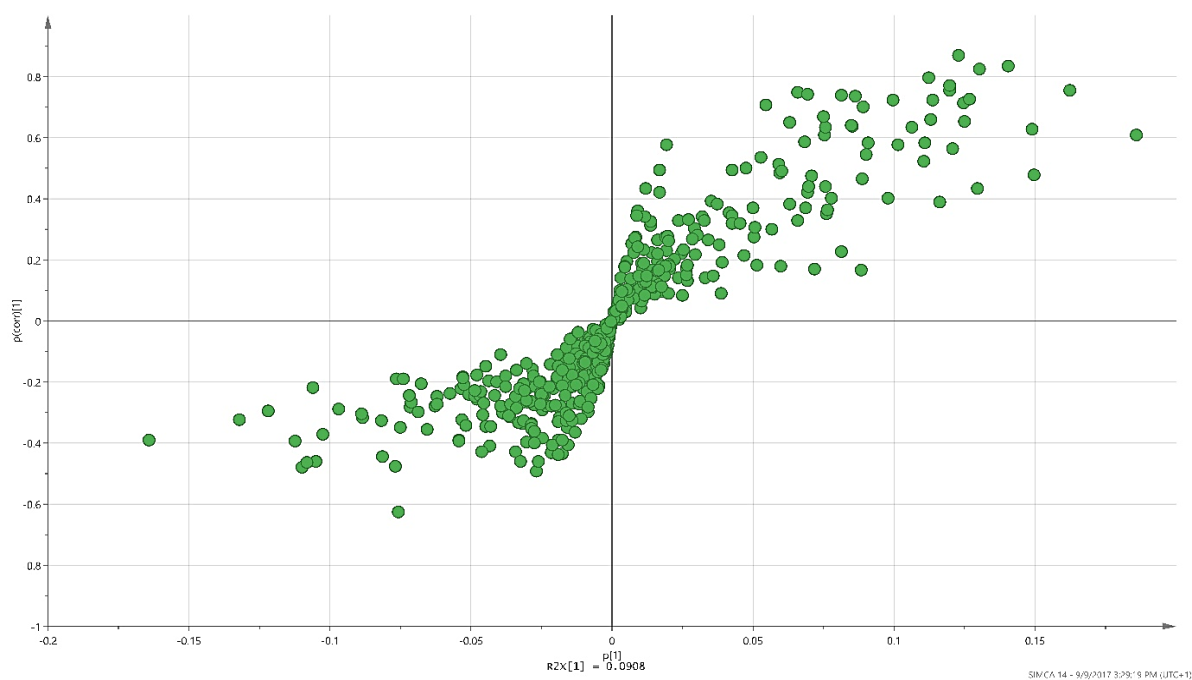


(c)


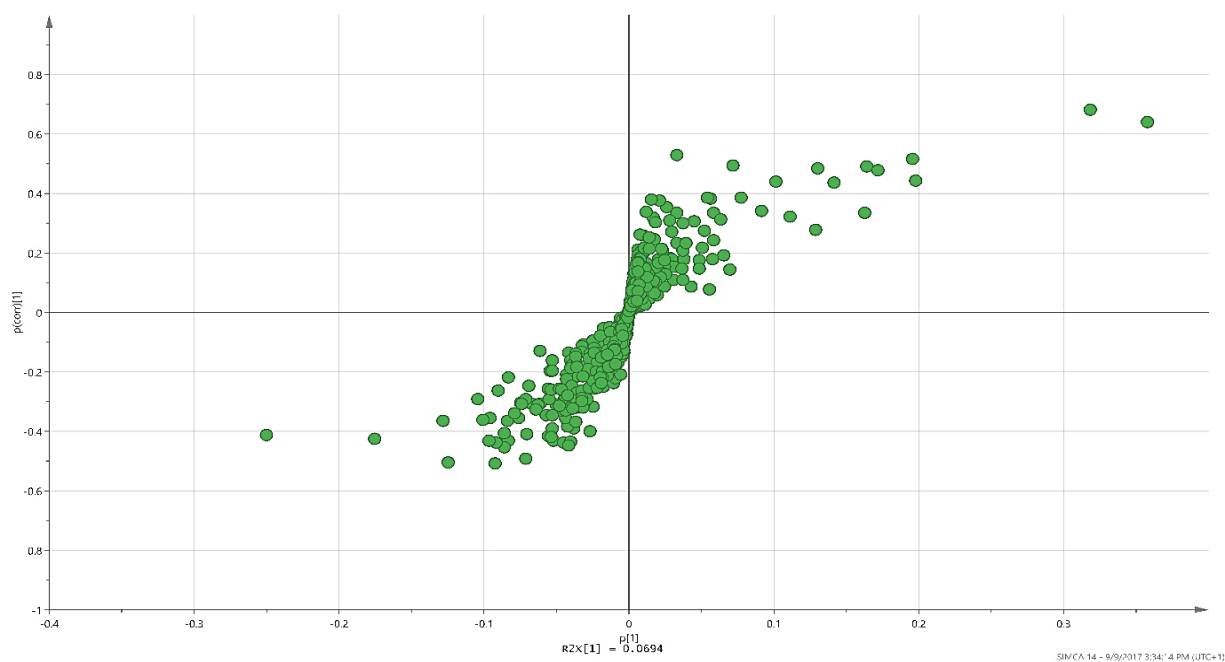


(d)

S9 – S-plots of (a) cod; (b) coley; (c) haddock and (d) whiting versus the other four species of fish under investigation identifying ions of significance which contribute to the separation of the five species of fish within the various chemometric models. Similar to that of the relevant ions identified within the pollock s-plot, all ions labelled in red are of great significance and contribute greatly to the separation of the five fish classes within the various chemometric models. Additionally, all ions have a VIP <2.
